# Maternal near miss in the intensive care unit: clinical and epidemiological aspects

**DOI:** 10.5935/0103-507X.20150033

**Published:** 2015

**Authors:** Leonam Costa Oliveira, Aurélio Antônio Ribeiro da Costa

**Affiliations:** 1 Postgraduate Education Unit in Mother and Child Health, Instituto de Medicina Integral Professor Fernando Figueira - Recife (PE), Brazil.; 2 Sector of Obstetrics and Gynecology, Instituto de Medicina Integral Professor Fernando Figueira - Recife (PE), Brazil.

**Keywords:** Pregnancy complications, Morbidity, Maternal mortality, Pregnancy, high risk, Pre-eclampsia, Intensive care units

## Abstract

**Objective:**

To analyze the epidemiological clinical profile of women with maternal near miss according to the new World Health Organization criteria.

**Methods:**

A descriptive crosssectional study was conducted, in which the records of patients admitted to the obstetric intensive care unit of a tertiary hospital in Recife (Brazil) over a period of four years were analyzed. Women who presented at least one near miss criterion were included. The variables studied were age, race/color, civil status, education, place of origin, number of pregnancies and prenatal consultations, complications and procedures performed, mode of delivery, gestational age at delivery, and maternal near miss criteria. The descriptive analysis was performed using the program Epi-Info 3.5.1.

**Results:**

Two hundred fifty-five cases of maternal near miss were identified, with an overall ratio of maternal near miss of 12.8/1,000 live births. Among these cases, 43.2% of the women had incomplete primary education, 44.7% were primiparous, and 20.5% had undergone a previous cesarean section. Regarding specific diagnoses, there was a predominance of hypertensive disorders (62.7%), many of which were complicated by HELLP (hemolysis, elevated liver enzymes, and low platelets) syndrome (41.2%). The laboratory near miss criteria were the most often observed (59.6%), due mainly to the high frequency of acute thrombocytopenia (32.5%).

**Conclusions:**

A high frequency of women who had a low level of education and who were primiparous was observed. According to the new criteria proposed by the World Health Organization, hypertensive pregnancy disorders are still the most common among maternal near miss cases. The high frequency of HELLP syndrome was also striking, which contributed to acute thrombocytopenia being the most frequent near miss criterion.

## Abstract (pt)

**Objetivo:**

Analisar o perfil clínico epidemiológico de mulheres com *near miss* materno segundo os novos critérios da Organização Mundial da Saúde.

**Métodos:**

Foi realizado um estudo descritivo, tipo corte transversal, analisando-se os prontuários das pacientes admitidas na unidade de terapia intensiva obstétrica de um hospital terciário do Recife (Brasil), em um período de quatro anos. Foram incluídas as mulheres que apresentavam pelo menos um dos critérios de *near miss*. As variáveis estudadas foram: idade, raça/cor, estado civil, escolaridade, procedência, número de gestações e consultas de pré-natal, complicações e procedimentos realizados, via de parto, idade gestacional no parto e critérios de *near miss* materno. A análise descritiva foi executada utilizando-se o programa Epi-Info 3.5.1.

**Resultados:**

Foram identificados 255 casos de *near miss* materno, totalizando uma razão de *near miss* materno de 12,8/1.000 nascidos vivos. Dentre esses casos, 43,2% das mulheres apresentavam ensino fundamental incompleto; 44,7% eram primigestas e 20,5% tinham realizado cesariana prévia. Quanto aos diagnósticos específicos, houve predominância dos distúrbios hipertensivos (62,7%), sendo que muitos deles foram complicados pela síndrome HELLP (41,2%). Os critérios laboratoriais de *near miss* foram os mais observados (59,6%), em função, principalmente, da elevada frequência de plaquetopenia aguda (32,5%).

**Conclusão:**

Evidenciou-se uma frequência elevada de mulheres com baixa escolaridade e primigestas. Com os novos critérios propostos pela Organização Mundial da Saúde, os distúrbios hipertensivos da gestação continuam sendo os mais comuns entre os casos de *near miss* materno. Destaca-se ainda a elevada frequência da síndrome HELLP, o que contribuiu para que a trombocitopenia aguda fosse o critério mais frequente de *near miss*.

## INTRODUCTION

According to the World Health Organization (WHO), a woman who experiences a life threatening situation and survives during pregnancy or childbirth or within 42 days of the end of pregnancy corresponds to a case of maternal near miss.^(1)^ The incidence of maternal near miss cases is higher than that of death, and these cases are currently considered a public health problem in Latin America.^(2)^ It is estimated that for every maternal death, there are on average 15 cases of near miss.^(3,4)^

Near miss is a better indicator of maternal health service quality than death is because near miss provides information that helps elucidate the factors that contribute to a fatal outcome; it is therefore used as a basis for the adoption of measures aimed at improving maternal care.^(5,6)^

Since the introduction of the near miss concept in 1991 by Stones et al.,^(6)^ numerous criteria have been used to characterize it.^(7-9)^ These criteria can be grouped as follows: clinical criteria related to a specific disease, criteria related to interventions or procedures, and criteria based on organ dysfunction.^(1)^ The maternal near miss ratio (number of near miss cases per 1,000 live births - LBs), when considering criteria based on signs and symptoms, is 27.8/1,000 LBs, higher than that based on organ dysfunction (10.2) or treatment (2.1) criteria, such as transfusion, intubation, surgery, or hemodialysis.^(10)^ In contrast, there is a greater tendency toward mortality when organ dysfunction criteria are adopted, with one case of maternal death for every six near miss cases. When signs and symptoms are used as the defining criteria, there is one case of death for every 35 cases of near miss.^(10)^

Given the heterogeneity of maternal near miss criteria and the necessity of standardizing them, in 2009, the WHO established new defining criteria (Table 1), which theoretically can be used at any hospital level, whether of low, medium or high complexity.^(1)^ The adoption of the WHO laboratory and treatment criteria to identify maternal near miss cases is a valid and effective method, as was concluded in a pre-validation study.^(11)^ That study also provided support for the recommendation to use such parameters throughout the world.^(11)^

**Table 1:** Maternal near miss diagnostic criteria according to the World Health Organization^(1)^

**Clinical**
Acute cyanosis
"Gasping" (terminal respiratory pattern in which breathing is laborious and audible)
Respiratory rate > 40 or < 6 breaths per minute
Shock (persistent severe hypotension, defined as SBP < 90mmHg for ≥ 60 minutes with a pulse of at least 120 beats per minute, despite liquid infusion [> 2L])
Oliguria unresponsive to fluids or diuretics (urine output < 30mL/hour for 4 hours or < 400mL/24 hours)
Coagulation disorders (coagulation failure as assessed by a clotting assay or by the absence of coagulation after 7 to 10 minutes)
Loss of consciousness for 12 hours or more (defined as a score < 10 on the Glasgow Coma Scale)
Loss of consciousness and absence of a pulse or heartbeat
Stroke (neurological deficit of cerebrovascular cause that persists for more than 24 hours)
Uncontrolled convulsion
Jaundice in the presence of pre-eclampsia (pre-eclampsia is defined as the presence of hypertension associated with proteinuria. Hypertension is defined as SBP ≥ 140mmHg and/or DBP ≥ 90mmHg on at least two occasions, with an interval of 4 to 6 hours after the 20^th^ week of pregnancy. Proteinuria is defined as excretion of 300mg or more protein in 24 hours, or ≥ 1 + proteinuria in at least two measurements with an interval of 4 to 6 hours)
**Laboratory**
SO_2_ < 90% for 60 minutes or more
PaO_2_/FiO_2_ < 200mmHg
Creatinine ≥ 300/*µ*mol/L or ≥ 3.5mg/dL
Bilirubin ≥ 100*µ*mol/L or ≥ 6.0mg/dL
pH < 7.1
Lactate > 5
Acute thrombocytopenia (< 50,000)
Loss of consciousness and presence of glucose and ketone in the urine
**Treatment**
Use of vasoactive drugs
Hysterectomy due to infection or hemorrhage
Transfusion ≥ 5 red blood cell units
Intubation and ventilation for ≥ 60 minutes unrelated to anesthesia
Dialysis for acute renal failure
Cardiorespiratory arrest

SBP - systolic blood pressure; DBP - diastolic blood pressure; SO_2_ - oxygen saturation; PaO_2_/FiO_2_ - arterial oxygen partial pressure/fraction of inspired oxygen.

Regarding the clinical and epidemiological profiles of women with maternal near miss, being over 35 years of age, being without a partner, primiparity, or having had a prior cesarean section (CS) are factors that have an independent association with the occurrence of maternal near miss. This was the conclusion of a multi-center study, published in 2010, that included 120 hospitals in eight countries across Latin America.^(2)^ A secondary analysis of data from a Brazilian demographic health survey, conducted in 2006 and 2007 and involving the five Brazilian geographical regions, showed that women over 40 years of age and with less than eight years of education are at a significantly higher risk of becoming a maternal near miss case.^(12)^ By contrast, no association was found between civil status, race, income, place of residence, or parity.^(12)^ These surveys were, however, conducted before maternal near miss criteria were defined by the WHO, and some of the criteria used therefore differed from those used currently.^(2,12)^

Given the high frequency of maternal near miss cases, its use as an indicator of maternal health, and the emergence of new defining criteria, this article aims to describe, according to the new WHO criteria, the clinical and epidemiological profile of patients with maternal near miss admitted to an intensive care unit (ICU) of a tertiary referral hospital in Recife, Brazil.^(1,13)^

## METHODS

This study was conducted after approval by the Research Ethics Committee (REC) of the *Instituto de Medicina Integral Professor Fernando Figueira* (IMIP) according to the principles governing research on human beings, Resolution 196/96 of the National Health Council (*Conselho Nacional de Saúde*), under number 2028-10 on November 19, 2010. Considering also that the study was retrospective and evaluated only data collected from medical records, the REC was asked to grant a waiver of the informed consent form.

The study was conducted at the IMIP, a charity located in Recife (Pernambuco, Brazil), which has an obstetric ICU with 12 beds exclusively for pregnant women and high-risk mothers and an annual hospitalization figure of close to 800 patients.

**Figure 1:**
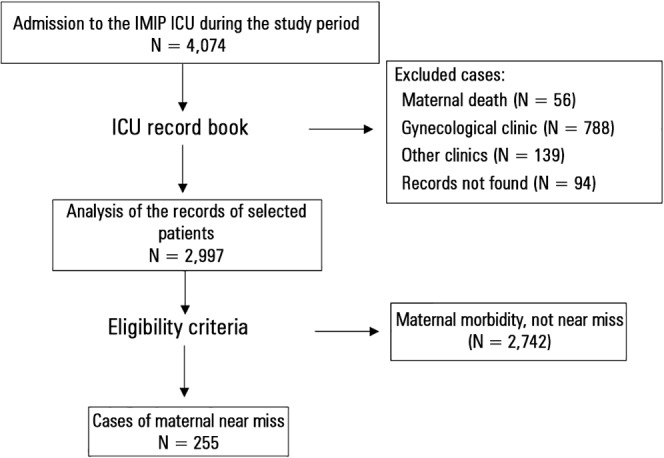
Flowchart of participant selection. ICU - intensive care unit; IMIP - *Instituto de Medicina Integral Professor Fernando Figueira*.

This investigation was a cross-sectional study that, based on the IMIP Obstetric ICU admissions and discharge records, identified 2,997 patients who were hospitalized during the pregnancy-childbirth cycle between January 2007 and December 2010. These patients’ records were requested from the hospital archives, and after analysis, those having at least one WHO-defined maternal near miss criterion were included in the study (Table 1), totaling 255 cases (Figure 1).

Data were collected by the principal investigator and two trained research assistants from the Pernambuco School of Health (*Faculdade Pernambucana de Saúde*) Course in Medicine, linked to the Institutional Program for Scientific Scholarships (*Programa Institucional de Bolsas de Iniciação Científica* -PIBIC).

The studied variables were age (expressed in complete years); race (categorical, polychotomous variable classified based on medical records data as white, black, mixed race); civil status (dichotomous nominal variable classified as with or without a partner); education (categorized as less than eight years of education or as eight or more years of education); place of origin (nominal variable, expressed as patients from Recife or from the inner state of Pernambuco or its metropolitan region); reproductive history (number of pregnancies and births, history of abortion, stillbirth, CS); preexisting conditions; prenatal care; diagnoses during hospitalization (severe pre-eclampsia, hypertension aggravated by pregnancy, eclampsia, HELLP [hemolysis, elevated liver enzymes, and low platelets] syndrome, postpartum hemorrhage, placental abruption, hemoperitoneum, sepsis, endometritis, pneumonia, pyelonephritis, acute renal failure, thrombocytopenia, or need for blood products transfusion or laparotomy); length of hospital stay; gestational age at delivery; delivery mode; and WHO maternal near miss criteria types.

Regarding the definition of maternal near miss criteria, those recommended by the WHO were adopted (Table 1). All data were entered in specific files created in Microsoft Excel, 2003 version. Statistical analysis was performed using the Epi-Info 3.5.1 program. For categorical variables, a descriptive analysis was conducted with frequency estimation and measures of central tendency, and their dispersions were calculated for quantitative variables.

## RESULTS

In total, 255 cases of maternal near miss were identified among the patients admitted to the IMIP obstetric ICU. During the study period, there were 19,940 LBs, yielding a maternal near miss ratio of 12.8/1,000 LBs. The length of hospital stay ranged from five to 86 days, averaging 14.8 ± 10.27.

The age of the participants was between 14 and 45 years, with a mean of 25.6 ± 6.99 years. Furthermore, 57.3% were mixed race, 64.3% had partners, 43.2% had less than eight years of education, and only 18.8% were from Recife (Table 2).

**Table 2:** Biological and socio-demographic characteristics and obstetric history of maternal near miss cases

**Characteristics**	**N (%)**
Age (years)	
14 - 19	55 (21.6)
20 - 34	170 (66.7)
35 - 45	30 (11.8)
Race/Color*	
Mixed race	146 (57.3)
White	45 (17.6)
Black	18 (7.1)
Civil status**	
With a partner	164 (64.3)
No partner	48 (18.8)
Education (years)*	
< 8	110 (43.2)
≥ 8	99 (38.8)
Place of origin	
Recife	48 (18.8)
MRR and inner state	207 (81.2)
Primiparous***	114 (44.7)
History of abortion	48 (18.8)
Previous CS (Yes)***	52 (20.5)

MRR - metropolitan region of Recife. Number of records without reports:

* 46,

** 43,

*** 12.

With respect to obstetric history, 44.7% of patients were primiparous, 18.8% had a history of abortion, and 20.5% had a history of previous CS (Table 2). The main diseases present prior to pregnancy were hypertension (6.7%), hematologic diseases (4%), heart disease (2.7%), asthma (2%), diabetes (2.4%), and epilepsy (1.2%). An analysis of prenatal care revealed that only 12 (4.9%) women did not attend prenatal care, and 121 (49.2%) had fewer than six visits. CS was the main delivery method, with 188 cases (76.4%). The median gestational age at delivery was 35 weeks, with 54.5% of births premature.

The main disorders presented by the study participants were hypertension (62.7%), hemorrhage (53.7%), infections (49%), heart disease (4.7%), and thromboembolism (2.4%). Among the 160 cases of hypertensive disorders, 108 (42.3%) were severe pre-eclampsia, 35 (13.7%) were eclampsia, and 17 (6.7%) were chronic hypertension exacerbated by pregnancy. One hundred five (41.2%) participants had HELLP syndrome.

The most common infectious disorder was endometritis (25.1%), followed by pneumonia (19.6%) and sepsis, which occurred in 16.9% of patients. Regarding bleeding disorders, there were 90 (35.3%) cases of postpartum hemorrhage, 29 (11.4%) cases of placental abruption, and four cases of placental accreta. There were also cases of uterine rupture (three) and placenta previa (one). Other complications or procedures performed on the participants were acute pulmonary edema (13.7%), blood transfusions (65.1%), central venous catheterization (18.8%), laparotomy (14.5%), and tracheostomy (2.4%).

Laboratory near miss criteria were present in 59.6% of participants, while clinical and treatment criteria occurred in 50.2% and 49% of cases, respectively (Table 3). Most patients (46.7%) were pregnant when presenting some of these criteria, and there was a median of only one criterion per patient.

**Table 3:** Maternal near miss criteria

	**N (%)**
Maternal near miss criteria	
Clinical criteria	128 (50.2)
Laboratory criteria	152 (59.6)
Treatment criteria	125 (49)
Time of occurrence of near miss	
Pregnancy	119 (46.7)
Labor/delivery	42 (16.5)
Postpartum	94 (36.9)
Number of criteria (Mean = 2.4/SD = 2.13)	
One	133 (52.2)
Two	47 (18.4)
Three	25 (6.7)
Four	17 (9.8)
Five to 12	33 (12.9)

The main maternal near miss clinical criteria included shock (17.6%), a respiratory rate exceeding 40 breaths per minute (bpm) (16.5%), and loss of consciousness for 12 hours or more (13.7%). The main laboratory criteria were platelet count below 50,000 (32.5%), creatinine above 3.5mg/dL (16.1%), and oxygen saturation lower than 90% for more than 60 minutes (10.6%). The most prevalent treatment criteria were tracheal intubation (23.5%), hysterectomy (20%), and transfusion of five or more units of packed red blood cells (19.2%) (Table 4). Clinical and laboratory criteria appeared mainly during pregnancy (42.2 and 57.9%, respectively), whereas treatment criteria occurred mainly postpartum (45.6%).

**Table 4:** Maternal near miss criteria presented by women admitted to the obstetric intensive care unit

**Maternal near miss criteria**	**N (%)**
Clinical	128 (50.2)
Shock	45 (17.6)
Respiratory rate > 40bpm	42 (16.5)
Loss of consciousness	35 (13.7)
Convulsion	19 (7.5)
Jaundice in the presence of preeclampsia	12 (4.7)
Oliguria	10 (3.9)
Absence of a pulse or heartbeat	10 (3.9)
Coagulation disorder	11 (4.3)
Cyanosis	8 (3.1)
Stroke	6 (2.4)
Laboratory	152 (59.6)
Platelets < 50,000	83 (32.5)
Creatinine > 3.5mg/dL	41 (16.1)
SO_2_ < 90%	27 (10.6)
Bilirubin ≥ 6mg/dL	19 (7.5)
PaO_2_/FiO_2_ < 200	18 (7.1)
Diabetic ketoacidosis	8 (3.1)
pH < 7.1	7 (2.7)
Treatment	125 (49)
Intubation and ventilation ≥ 60 minutes	60 (23.5)
Hysterectomy	51 (20)
Blood transfusion ≥ 5IU of red blood cells	49 (19.2)
Vasoactive drugs	18 (7.1)
Dialysis	15 (5.9)
Cardiopulmonary resuscitation	10 (3.9)

bpm - breaths per minute.

The indicators proposed by the WHO to monitor the quality of obstetric care are the mortality index (number of maternal deaths divided by the number of women with life threatening conditions, expressed as a percentage) and the maternal near miss/maternal mortality rate (proportion of maternal near miss to maternal death cases).^(1)^ In this study, these indicators were 18% and 4.5:1, respectively, with 56 maternal deaths among the patients admitted to the IMIP obstetric ICU during the study period.

## DISCUSSION

The incidence of maternal near miss described in the literature varies widely, from 0.7 to 101.7 cases per 1,000 births.^(10,14,15)^ In a Brazilian demographic health survey of more than 5,000 women conducted in 2006 that adopted the Mantel and Waterstone criteria, this ratio was 21.1/1,000 LBs, above that found in our study, which was 12.8/1,000 LBs.^(12)^ This difference is due in part to the different criteria used.^(10)^ With the standardization of maternal near miss criteria proposed by the WHO, this wide variation can be reduced, thus allowing more reliable comparisons.^(1)^ In two other Brazilian studies that adopted the new WHO classification, the ratio was 9.35 and 13.5/1,000 LBs, which was close to the result found in our study.^(11,16)^ Notably, in calculating the maternal near miss rate, the WHO recommends using LBs as the denominator and not the number of births, as reported in previous studies.^(1)^

Some studies characterize maternal near miss as a problem of women at more advanced ages, who are single and primiparous.^(8,12,16)^ In this study, the percentage of patients over 35 years of age (11.8%), without partners (18.8%), and primiparous (44.7%) was similar to that found in a multicenter study of nearly 3,000 cases (14.1, 23.6, and 36%, respectively).^(2)^ The same study also identified an independent and positive association between these three factors and the occurrence of near miss.^(2)^

In another database-based retrospective Brazilian study, which included more than 5,000 women from the five regions of the country, the risk of maternal near miss was nearly twice as high among women who were over 40 years of age and had a low level of education.^(12)^ In the present study, the frequency of women with less than eight years of education (43.2%) was slightly lower than that found in that study (54.5%).^(12)^ It therefore appears that there is a genuine trend toward a greater association of near miss among older, single, primiparous, and poorly educated women.

Although an association between maternal near miss and place of residence was not demonstrated, our study draws attention to the high percentage of women from municipalities in the interior of the state.^(12)^ This finding most likely reflects the precariousness of care in these locations and highlights the need for improvements, which may be achieved by organizing regional care and decentralizing prenatal and high-risk delivery care, as well as providing these regions with equipment, laboratory tests, drugs, and specialized professionals.

The percentage of patients who did not receive prenatal care was low-lower than that found in a retrospective study conducted in Recife in 2007 (9.7%).^(17)^ This finding demonstrates the expansion of prenatal care and suggests an improvement in reaching the population. However, it suggests a low quality of these services because, although the majority of women had access to prenatal consultations, almost half of them suffered near miss. Researching only the number of consultations might have been inadequate when evaluating prenatal care; other variables such as the timing of the onset of prenatal care, the number of tests performed, and proper completion of pre-natal records, may be used in the future.

In a retrospective and observational study in Italy with more than 1,200 cases of maternal near miss and another study by the WHO that was cross-sectional and multicentric with nearly 3,000 cases, CS was the main delivery mode, with a frequency of 70% and 59.5%, respectively. This is similar to the findings of the present study (76.4%).^(2,18)^ Some authors consider CS a factor that increases the odds of a woman becoming a case of near miss by up to five times; however, this association may be affected by confounding factors.^(19,20)^ Whether CS is a risk factor for near miss, or whether it is actually a consequence of this condition, is still therefore unclear.^(2,20)^ Another multicenter study also postulates that high rates of CS may be acceptable among such patients, due to the urgency in resolving the pregnancy and unfavorable cervical or fetal conditions.^(19)^

By contrast, CS cause repercussions in a subsequent pregnancy and is recognized as an independent risk factor in maternal morbidity.^(21)^ In our study, CS in a previous pregnancy was 20.5%, very close to that found in a WHO survey (18.2%), which showed an independent and positive association between previous CS and the occurrence of near miss (adjusted OR = 1.63; 95% CI = 1.47 to 1.81).^(2)^

Severe preeclampsia was the main diagnosis associated with near miss, coinciding with the findings of other Brazilian studies.^(11,17,19)^ However, this finding differs from those in studies conducted in developed countries, in which hemorrhage is ranked first.^(19,22)^ The main cause of maternal death in Brazil is also hypertensive disorders, which indicates the seriousness of these situations and their contribution to the morbidity and mortality of women in pregnancy and childbirth.^(23)^

Bleeding disorders are the second most common complication among cases of near miss, and the procedure most often performed is blood transfusion (65.1%). These findings are similar to those of a Dutch study (66.2%) and a study conducted in Recife (66.2%).^(17,24)^ This finding may be explained by the numerous risk factors for postpartum hemorrhage presented by patients with near miss, such as preeclampsia, CS, and intrauterine infection, among others.^(25,26)^

Sepsis occurred in 16.9% of patients, but in another Brazilian study, this result was 23.7%.^(27)^ Although this disease is not one of the most frequent complications, many studies consider it to have a higher mortality rate (7.4%), surpassing hemorrhagic (2.8%) and hypertensive (0.4%) disorders.^(28,29)^

Regarding near miss criteria, the most frequent were laboratory criteria, followed by clinical and treatment criteria. In a five-year retrospective study of 194 near miss cases (in the city of Campinas, Brazil) that adopted only laboratory and treatment criteria, the most common were treatment criteria, with almost half of the near miss patients requiring mechanical ventilation (49.6%).^(11)^ In our study, mechanical ventilation was required in less than one-quarter of cases. This finding may be attributed to local differences in accessibility to resources and interventions,^(1)^ and is one of the drawbacks of criteria based only on treatment because they require a more complex hospital and laboratory structure.^(1)^

Among all the maternal near miss criteria, the most prevalent was acute thrombocytopenia, possibly due to the high frequency of HELLP syndrome. Acute thrombocytopenia is known to increase the risk of maternal death, and a delay in its recognition may expose women to life-threatening conditions.^(30)^ The platelet count is an inexpensive and accessible test and can be requested for the early identification of such cases.

The main clinical maternal near miss criteria observed were shock, respiratory rate exceeding 40bpm, and loss of consciousness for 12 hours or more. These characteristics differ from those observed by Morse, who found that oliguria, shock, and coagulation disorder were the most frequent.^(16)^ Studies with larger samples are necessary for possible comparisons. One advantage of these criteria is that they do not require a highly complex laboratory or hospital structure and can be applied in low-complexity hospital units. Notably, in a validation study of WHO criteria to define maternal near miss, clinical criteria were not tested, and only treatment and laboratory criteria were validated.^(11)^ The same investigation also indicates a need for specific studies to validate clinical criteria.^(11)^

Premature delivery occurred in more than half of the study participants. This high rate of prematurity, also observed in a study conducted in Campinas (65%), may be explained by the severe conditions experienced by these patients, which can compromise the fetus and in most cases require immediate resolution.^(17)^

The use of indicators that facilitate comparisons between services, countries, and time may be associated with the study of maternal near miss.^(1)^ The mortality rate and proportion of near miss cases and maternal deaths were high in this study. There was one maternal death for every 4.5 cases of maternal near miss, indicating that many women with life-threatening conditions die; therefore, improvements to care during pregnancy, childbirth or the postpartum period are still necessary.^(30)^ To achieve this goal, it is important to study women who were near death but survived and to study maternal near miss.^(1,30)^

Study limitations include the fact that a prospective follow-up of patients, which might have yielded more information and decreased losses, did not occur. In addition, the fact that the data were collected from medical records did not allow some near miss criteria (such as gasping, presence of cyanosis, and measurement of lactate concentration) to be identified. However, the study design allowed the inclusion of a reasonable number of near miss cases, a subject of public health interest, directly linked to the reduction of maternal deaths.

This study helped to identify and characterize the population of women admitted to the obstetric ICU of the IMIP who experienced maternal near miss, which is a first step in helping to identify the factors related to these life-threatening situations. Multicenter studies involving a greater number of cases and adopting the criteria established by the WHO for maternal near miss can go further and lead to the development of preventive measures.

## CONCLUSIONS

This study showed that, among patients with maternal near miss, there is a high frequency of women who have a low level of education, who are primiparous, and who have had a previous cesarean section. The present investigation also determined that the most frequent near miss criteria were of the laboratory type and that there was a high prevalence of premature births among these women. In addition, with the adoption of the new World Health Organization maternal near miss criteria, our study revealed that hypertensive pregnancy disorder is still the most frequent diagnosis among such cases.
